# Role of Jumpstart Nutrition^®^, a Dietary Supplement, to Ameliorate Calcium-to-Phosphorus Ratio and Parathyroid Hormone of Patients with Osteoarthritis

**DOI:** 10.3390/medsci7120105

**Published:** 2019-11-22

**Authors:** Apurba Ganguly

**Affiliations:** Department of Research and Development, OPTM Research Institute, 145 Rashbehari Avenue, Kolkata 700029, India; apurbaganguly15@gmail.com; Tel.: +91-983-038-9616

**Keywords:** osteoarthritis, vitamin-K_2_, coenzyme-Q_10_, calcium-to-phosphorus ratio, parathyroid hormone

## Abstract

The aim of this study was to use Jumpstart Nutrition^®^ bone supplementing combination with vitamin-K_2_ and coenzyme-Q_10_ characterized by an innovative delivery system that improves bioavailability of calcium-to-phosphorus ratio (CPR) and parathyroid hormone (PTH) in the management of osteoarthritis (OA). This eight-week registry included 108 patients treated for symptomatic OA confirmed with radiological images. On top of that, 63 patients used Jumpstart Nutrition^®^ supplement, mainly prepared with calcium, phosphorus, coenzyme-Q_10_, vitamin-K_2_, vitamin-D_2_, vitamin-C, folic acid, curcumin and boswellic acids. Rescue medication was also recommended. Patients’ pain and functional capacity through outcome measures—knee-injury osteoarthritis outcome scale (KOOS) and Karnofsky performance scale (KPS), biomarkers such as levels of CPR, PTH and 25-hydroxy-vitamin-D were evaluated for the groups with and without supplement using appropriate kits. After eight weeks, the levels of CPR and PTH were all significantly improved (*p* < 0.001), fewer subjects had to use rescue medication (*p* < 0.05) and variation of pain and functional capacity under KOOS and KPS (*p* < 0.05) of the patients in the supplement group compared to controls. This registry study indicates that Jumpstart Nutrition^®^ can be used safely for effective management of OA patients for the amelioration of CPR, PTH and functional activities confirmed with biomarkers and radiological images correlated with the Kellgren-Lawrance scale.

## 1. Introduction

Osteoarthritis (OA) is an inflammatory disease that involves painful bone degeneration, muscles and connective tissues damage, which occurs around the world [1,2,3,4]. According to the Global Burden of Disease Study reports of the World Health Organization (WHO), nearly 130 million people will suffer from OA worldwide by 2050 and the number of women will be almost double that of the number of men aged over 60 years, constituting a significant social burden [5]. Several biochemical parameters are altered abnormally in various tissues, viz. blood, cartilages, bones and synovial fluid, during osteoarthritis [6,7,8,9].

Osteoarthritis is generally managed with the help of palliative measures and symptomatic medications that focus on the reduction and control of symptoms including lifestyle changes, weight control, exercise, administration of anti-inflammatories and analgesics along with calcium and vitamin D supplements to control vitamin and mineral deficiencies during OA [10,11].

Calcium and phosphorus are the most and second most abundant micronutrients that regulate neuromuscular function and skeletal mineralization in the body [12].

Our bones contain nearly 99% calcium, which regulates vascular contraction, vasodilatation, glandular secretion, muscular contraction, glycogen metabolism, neurotransmission and, finally, the maintenance of bone health and mineralization [13,14,15,16]. At the same time, 85% of phosphorus contained in the bones and teeth facilitates mineral metabolism, cellular signal transduction, the exchange of energy and, along with calcium, bone development [13,14,15,16]. A high level of phosphates, a form of phosphorus, in the blood is called hyperphosphatemia and hypophosphatemia occurs when the level of phosphates is too low in the blood. These symptoms include joint pain, muscle pain, muscle weakness, fatigue and low energy levels. The remaining 1% of the calcium is extracellular and plays a role in nerve conduction, muscle contraction, blood clotting and immune system activation [13,14,15,16,17]. Therefore, a decrease in blood calcium level causes bone deformation, renal disease and hypoparathyroidism, among other problems [18], and the estimated blood calcium-to-phosphorus ratio (CPR) is known to be a suitable marker during bone formation [14,19]. The ratio between the calcium and phosphorus in bone formation and their interaction during absorption and metabolism and the relationship between the consumption of these nutrients as supplements is the main objective of this study and their ratio is suggested to be maintained as 1.4:1.0 [20,21]. Kemi et al. has emphasized that a lower calcium-to-phosphorus ratio affects serum parathyroid hormone concentration and calcium metabolism in healthy people with adequate calcium intake [20]. Parathyroid hormone (PTH) is an 84-amino-acid peptide secreted by two pairs of parathyroid glands located adjacent to the back of the thyroid gland in the neck. PTH has two major mechanisms for promoting the absorption of calcium—by increasing Ca^2+^ reabsorption in the kidneys and by stimulating the activation of vitamin D, when the concentration of Ca^2+^ drops, the parathyroid gland releases PTH to help bring the Ca^2+^ concentration backup [22,23].

Although the effects of vitamin D metabolites on bone are complicated, it causes bone resorption by mature osteoclasts and regulates bone proteins. Vitamin D is a secosterol hormone that is present in humans in an endogenous (vitamin D_3_) and exogenous (vitamin D_2_) form at a ratio of approximately 2:1 [23,24]. Vitamin D_3_ (cholecalciferol) is synthesized in the skin under the influence of ultraviolet radiation. Vitamin D_3_ is also available in oral supplements and injection. Vitamin D_2_ (ergocalciferol) is produced by ultraviolet irradiation of the plant sterol ergosterol and is available through the diet. Both forms of vitamin D require further metabolism to be activated and their respective metabolism is indistinguishable [23,24]. The Institute of Medicine (IOM) (Tribhuvan University, Kathmandu, Nepal) has suggested that a 25(OH) vitamin D >20 ng/mL is adequate [25], while The Endocrine Society suggests that >30 ng/mL is optimal [26]. The IOM suggests that supplements of 600–800 IU daily will produce adequate levels in most adults, with an upper safe dose of 4000 IU daily [26]. Therefore, 8 µg of vitamin D has been mixed in the present supplement per serving dose of 25 g. 

Besides vitamin D, two other vitamins, namely vitamin-K_2_, known as the coagulation vitamin [27], and coenzyme-Q_10_ are absolutely required to bind calcium to make the skeleton stronger and less susceptible to fracture [28] and for muscle dysfunction associated with structural and alterations of skeletal muscle mitochondria, which controls the metabolism of reactive oxygen species (ROS), Ca^2+^ homeostasis and apoptosis [29].

Moreover, oral supplement of oleo-gum resin of *Boswellia serrata* (also called Indian frankincense or Salai guggal) and curcumin have been used as Ayurvedic medicine in India for centuries as a remedy for the treatment of chronic inflammatory diseases, including osteoarthritis and chronic bowel diseases [30,31,32,33]. 

At the same time, the bioactive substances of whey/soy protein such as hormones, growth factors and cytokines regulate cell growth in both normal cells and tumor cells by suppressing proliferation or enhancing apoptosis [34,35].

Several bone supplements available in the market add calcium and vitamin D, or folic acid, or a number of amino acids, or with hydrolyzed amino collagen, or with glucosamine. A bone supplement with vitamin-K_2_, coenzyme-Q_10_, boswellic acids and nano-curcumin (a powerful antioxidant) in addition to proportionate calcium, phosphorus, vitamin C mixed with protein powers of soy and whey is a first time endeavor to control CPR and PTH and to reduce high inflammatory and oxidative values in the management of OA.

The aim of the present supplement registry study was to conduct a pilot assessment for the improvement of bioavailability of calcium-to-phosphorus ratio (CPR), parathyroid hormone (PTH) and vitamin D in the management of OA. 

## 2. Materials and Methods

### 2.1. Recruitment of Patients

This eight-week registry included 243 patients, aged 40 to 75 years old, who were treated at Organic Phyto Therapeutic Method (OPTM) Health Care (P) Ltd. (a government registered clinic with license number 34218956, Kolkata, West Bengal, India) from November 2018 to January 2019. The OPTM Research Institute Ethics Committee has evaluated and approved the present study protocol. The institute is also registered with the government. An Institutional Review Board approved the consent form for the physical examinations, blood sample collections and radiological images, which was required for the study, and it was signed by all patients in the first phase of the screening procedure.

### 2.2. Exclusion Criteria

One hundred and 35 of 243 patients with concomitant diseases or risk conditions requiring drug treatment, severe metabolic disorders, drugs/alcohol addiction and /or psychiatric diseases, oncological conditions, pregnant, planned conception, multiple drug dependence, a history of cancer including caranomatosis and granulocytic leukemia, a history of chronic liver, kidney and heart diseases, and also patients who did not agree to a physical evaluation and /or attend weekly follow-up visits were all included in the exclusion criteria.

### 2.3. Study Design

**Figure 1:**
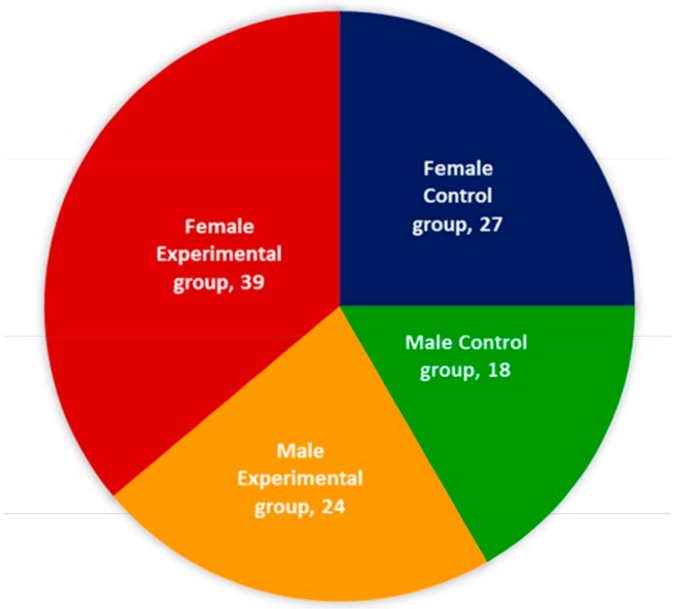
Gender-wise allocation of 63 experimental subjects and 45 control subjects.

After evaluating the exclusion criteria, 63 of the remaining 108 patients suffering with acute OA, who had elevated levels of CPR and PTH confirmed with radiological images and biochemical parameters, were treated with Jumpstart Nutrition^®^ bone supplement and the remaining 45 subjects were treated without supplements and considered as control subjects. The baseline demographic characteristics of all patients are presented in Table 1. The gender-wise classifications for both the groups are shown in Figure 1. 

### 2.4. Evaluation of Knee-Injury Osteoarthritis Outcome Scale

Pain and disability are common factors in any OA patient. Therefore, the knee-injury osteoarthritis outcome scale (KOOS) developed by Roos et al. [36] to assess the patient’s opinion about their knee pain and associated problems as an instrument. All the scoring data under KOOS were evaluated for each patient at the baseline and at the end of the eight-week treatment for both the groups. Their mean, standard deviation, and percentage of improvement and declination were also graphically evaluated separately by gender for both the groups.

### 2.5. Evaluation of Karnofsky Performance Scale

The Karnofsky performance scale (KPS) was used to determine a patient’s prognosis to carry out daily activities and it ranges from zero to 100 [37]. A higher score shows the patient is in a better position to carry out daily activities. The mean, standard deviation, and the percentage of improvement and declination of performance were separately evaluated at the end of the registry period compared to the baseline activities for both the groups. 

### 2.6. Evaluation of Biochemical Parameters

From each subject of the with and without supplementary groups, 5 mL of blood sample was collected in a vial (vial containing ethylenediaminetetraacetic acid, EDTA). Then blood samples were centrifuged at 1000×g for 10 min at 4 °C (Cryo Scientific Systems Pvt. Ltd., Chennai, Tamil Nadu, India) to obtain serum. Finally, the serum was used to analyze calcium, phosphorus, PTH hormone, and 25-hydroxy vitamin-D for both the groups. A kit Calcium AS FS was used to carry out the quantitative assessment of calcium (mg/dL) by the method of the photometric test using Arsenazo III (DiaSys Diagnostic Systems GmbH, Holzheim, Germany) at a wavelength of 650 nm. The kit was developed from the methods of Michaylova and Ilkova [38] and Bauer [39]. The quantitative assessment of phosphorus (mg/dL) was carried out using the photometric test (DiaSys Diagnostic Systems GmbH) at a wavelength of 340 nm. The kit was developed from the methods of Thomas [18]. A kit Intact-PTH ELISA (enzyme-linked immunosorbent assay) (Biomerica Inc., Irvine, CA, USA, Ref 7022) was used to carry out the quantitative assessment of PTH (pg/mL) as an immunoassay. The kit was developed from the methods of Raisz et al. [40] Mallette [41] and Kruger et al. [42]. Using an ELISA kit, 25-hydroxy vitamin D was measured; this method first described by Engvall in 1972 [43]. Each test for each patient was rechecked by the BS-240 Mindray fully automated biochemistry analyzer before reporting the final test results for both the groups. The mean, standard deviation, and their mean differences (MDs), 95% confidence intervals (CIs), *p*-values, odds ratio and their 95% CIs and coefficient of variances were evaluated for each patient at the baseline and at the end of the eight-week registry period.

### 2.7. Evaluation of Pearson’s Correlation Coefficients between Two Biomarkers

To determine the predictive values of each abnormal level of biomarker at the baseline compared to the level at the end of eight-week registry period, Pearson’s correlation coefficients were evaluated with their *p*-values.

### 2.8. Evaluation of Radiological Images with the Kellgren-Lawrance Grading Scale

Radiological images for both knee joints of 108 patients for both the groups were evaluated at the baseline and at the end of the eight-week registry periods. The anterior-posterior (AP) views of the knee joints of two female and two male patients of both groups were separately evaluated. The Kellgren and Lawrance (KL) system is a common method of classifying the severity of knee osteoarthritis, developed by Kellgren et al. in 1957 [44]. The KL classification was originally described using anterior-posterior (AP) knee radiographs based on the five grades.

### 2.9. Management of Supplement Studies

The formulation of the Jumpstart Nutrition^®^ bone supplement has been scientifically evaluated by Nanophyto wellness Pvt Ltd, Kolkata, India. Food Safety and Standards Authority of India (FSSAI) has strictly scrutinized the pharmacokinetics of the ingredients used the supplement along with their dietary reference intakes (DRIs) as recommended by the Food and Nutrition Board of the Institute of Medicine, National Academy of Sciences (Washington, DC, USA). Finally, they have issued a license bearing the number 10018031002579 to the company under the Food Safety and Standards Act, 2006. 

Twenty-five grams of supplement was to be consumed, mixed with cold/warm water, preferably in the afternoon. The ingredients used in the supplement are: i) 737 mg of minerals composed of calcium, phosphorus, and iron in the ratio 5:4:0.21; ii) 125.13 mg of vitamins containing 100 mg coenzyme- Q_10_, 25 mg vitamin-C, 100 µg folic acid, 20 µg vitamin-K_2_, and 8 µg vitamin-D_2_; and iii) 275 mg of other phytonutrients such as boswellic acids and curcumin in the ratio 8:3 mixed with protein powers of soy and whey in the ratio 3:7, based on Recommended Dietary Allowance (RDA) guidelines.

The minerals were collected from Mitushi Biopharma (Ahmedabad, Gujarat, India), vitamins were collected from Herbo Nutra (Razapur Khurd, New Delhi) and other phytonutrients were collected from Sami Labs Ltd (Bangalore, India), and protein powders were collected from Kiwi Nutritech (Chennai, India).

The aim of the supplement studies is to define the field of active evaluation of supplements and the possible activation of proper balancing of the levels of calcium, phosphorus, vitamin-D, parathyroid hormone or of other management plans. 

The following rules were adopted for the use of supplements in this study:

- As the supplements are not drugs, they are not prescribed but are recommended for the improvement of the levels of calcium, phosphorus, vitamin D, and parathyroid hormone during the management of OA.
- The supplement is used in addition to the best management/ care, if available, based on appropriate international guidelines.
- There is no interference with any other treatment or preventive measure while using the supplement.
- A register is always maintained for the evaluation of these studies.
- The supplement is not very costly and easily available on the market without prescription.
- The patients considered as experimental subjects were those who have consumed the supplement continuously for eight weeks as per register.
- A possible placebo effect is explained, and no placebo is used.
- Safety and tolerability were strictly evaluated.

### 2.10. External Study Reviewers

All results and data before and after the treatment were evaluated by an external reviewing panel, not in contact with the registry patients.

### 2.11. Statistical Analysis

Continuous variables, such as serum CPR, PTH, and vitamin D levels, in the OA cohort are expressed as the mean, standard deviation (SD), and 95% CIs of differences and the experimental and control groups were compared using the Mann-Whitney U test, as the data do not follow a normal distribution. Non-parametric odds ratios (ORs) for CPR, PTH, and vitamin D, and their 95% CIs were performed to evaluate the accuracy of various measurements in predicting the experimental group compared to the control group. The statistical software IBM SPSS (version 20) was used for all statistical analyses. An alpha level of 5% was established, that is, a *p*-value less than 0.05 was considered statistically significant.

## 3. Results

### 3.1. Enrolment and Baseline Characteristics of Patients

Table 1 shows the baseline characteristic features of the total of 108 patients who were included in the study. All the patients were suffering with OA and had elevated levels of CPR, and PTH and a diminished level of vitamin D, along with bone erosion and skeletal muscle damaged confirmed by biochemical parameters and radiological images. Of these, 63 patients used Jumpstart Nutrition^®^ bone supplement. Gender-wise analysis of all the patients is shown in Figure 1.

### 3.2. Biochemical Parameters

**Figure 2:**
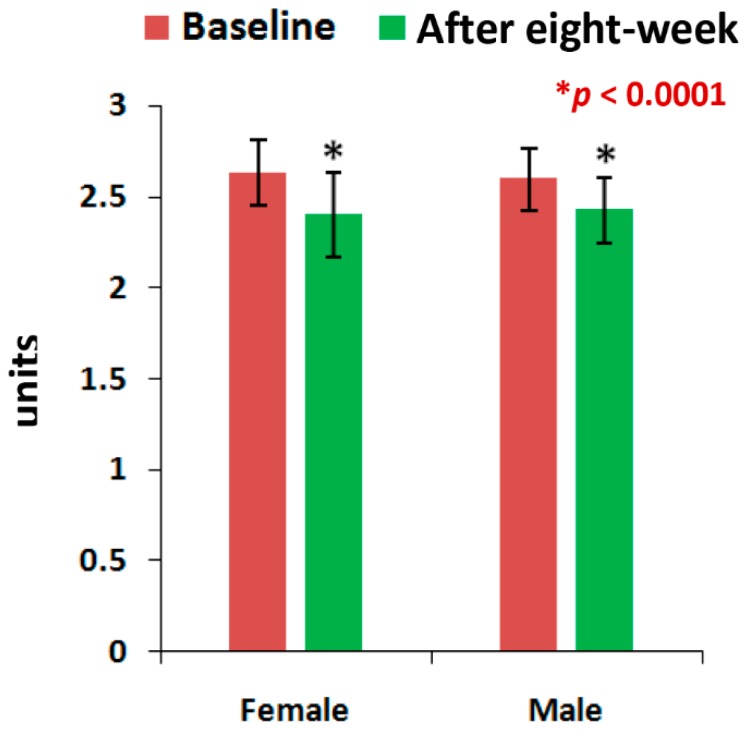
Levels of Calcium-to-Phosphorus ratio of 63 experimental subjects, female (*n* = 39) and male (*n* = 24), before and after eight-week using supplement (*p < 0.0001).

**Figure 3:**
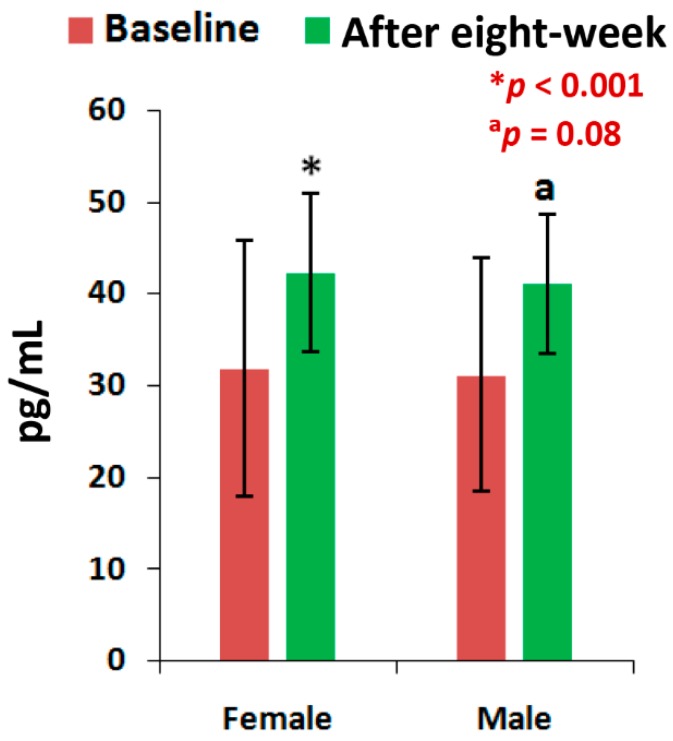
Levels of Parathyroid hormone of 63 experimental subjects, female (*n* = 39) and male (*n* = 24), before and after eight-week using supplement (*p < 0.001, ^a^*p* = 0.08).

**Figure 4:**
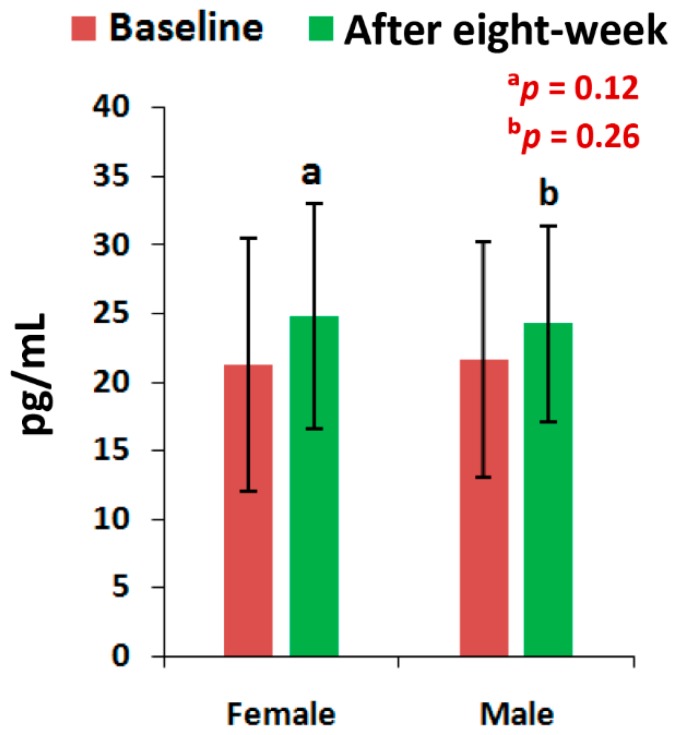
Levels of Vitamin-D of 63 experimental subjects, female (*n* = 39) and male (*n* = 24), before and after eight-week using supplement (^a^*p* = 0.12, ^b^*p* = 0.26).

**Figure 5:**
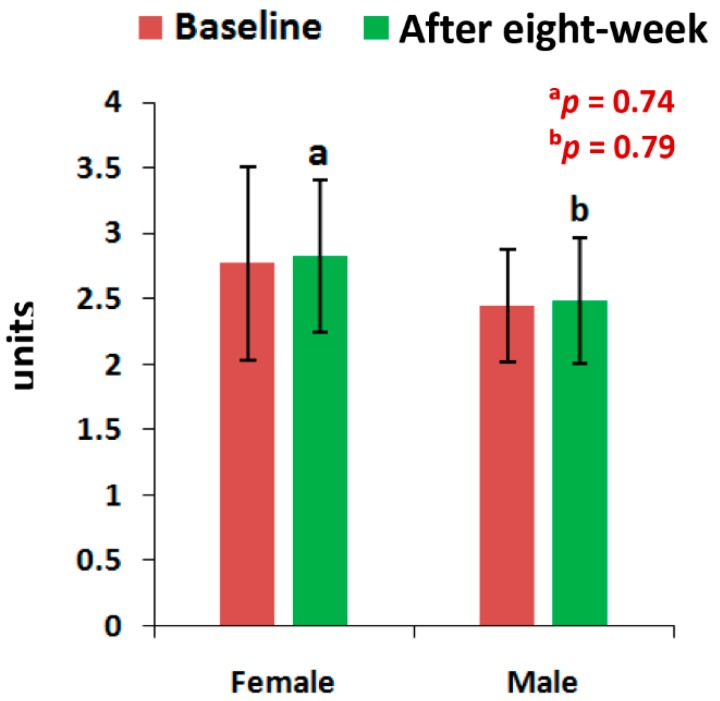
Levels of Calcium-to-Phosphorus ratio of 45 control subjects, female (*n* = 27) and male (*n* = 18), before and after eight-week without using supplement (^a^*p* = 0.74, ^b^*p* = 0.79).

**Figure 6:**
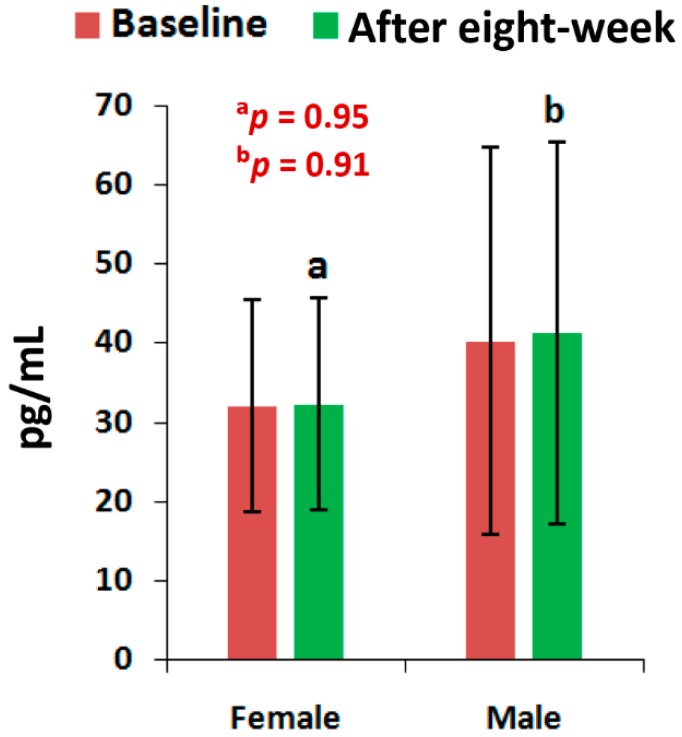
Levels of parathyroid hormone of 45 control subjects, female (*n* = 27) and male (*n* = 18), before and after eight-week without using supplement (^a^*p* = 0.95, ^b^*p* = 0.91).

**Figure 7:**
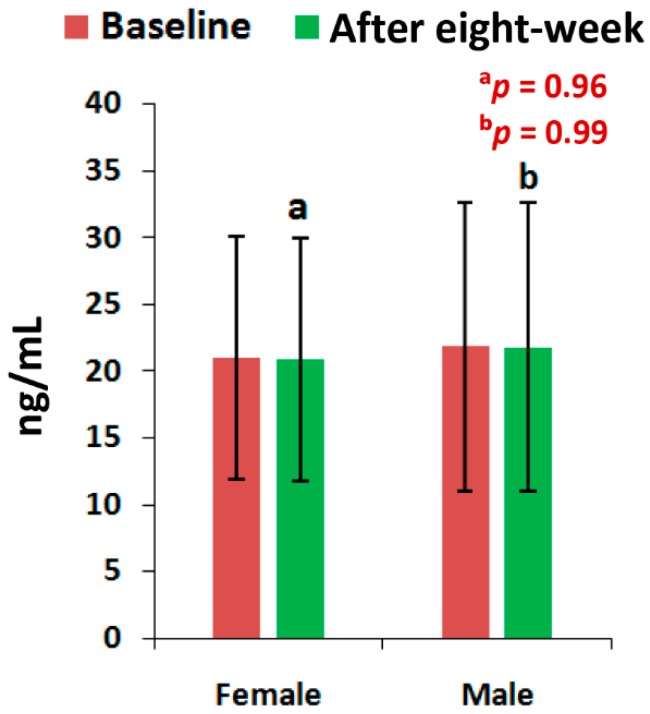
Levels of Vitamin-D of 45 control subjects, female (*n* = 27) and male (*n* = 18), before and after eight-week without using supplement (^a^*p* = 0.96, ^b^*p* = 0.99).

The mean levels of CPR, and PTH at the end of eight-week of treatment with the supplement were all highly significant (*p* < 0.05), except the level of PTH for men and the vitamin D levels of all patients, when compared to the baseline separately by gender (Figure 2, Figure 3 and Figure 4). All the biochemical levels of control subjects without supplement were not significant compared to the control (Figure 5, Figure 6 and Figure 7). 

Table 2 shows the levels of correlation coefficients between the ratio of calcium-to-phosphorus at the baseline (CPR^b^) and at the end of eight-week treatment with supplement (CPR^t^), were negatively correlated but not significant (*p* = 0.286 for females and *p* = 0.346 for males) while that of the levels of PTH and vitamin D were all highly significant (*p* < 0.01) for both female-only and male-only. The correlation coefficients between all the biochemical parameters of the control subjects at the baseline and at the end of eight-weeks without supplement for female-only patients and male-only patients were not significant. 

Table 3 shows that the percentage of co-efficient of variances for all the studied biochemical parameters of the experimental subjects with supplements were substantially reduced compared to the control subjects without supplements. 

### 3.3. Pain, Stiffness, Performance Parameters

**Figure 8:**
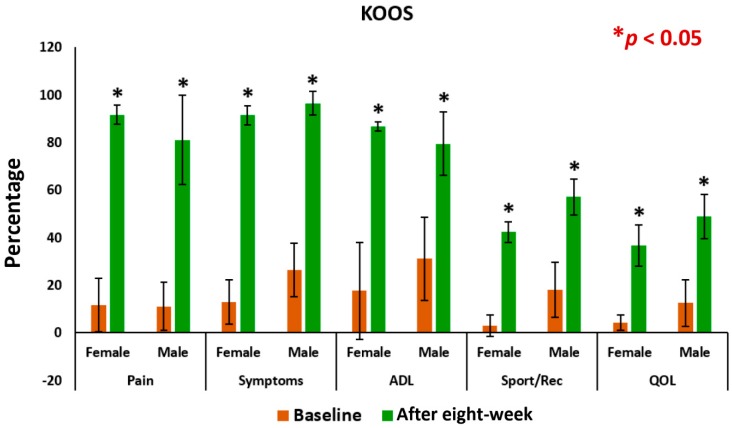
Analysis of knee-injury osteoarthritis outcome scale of 63 experimental subjects, female (*n* = 39) and male (*n* = 24), before and after eight-week with supplement (*p < 0.05), ADL: Activities of daily living; QOL: Quality of life.

**Figure 9:**
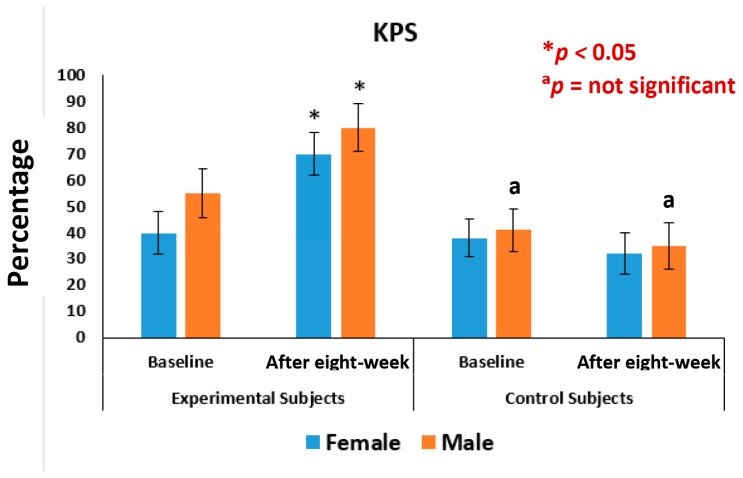
Analysis of Karnofsky performance scale of 63 experimental subjects, female (*n* = 39) and male (*n* = 24), and 45 control subjects, female-(*n* = 27) and male-(*n* = 18), before and after eight-week with and without supplement (*p < 0.05, ^a^*p* = not significant).

**Figure 10:**
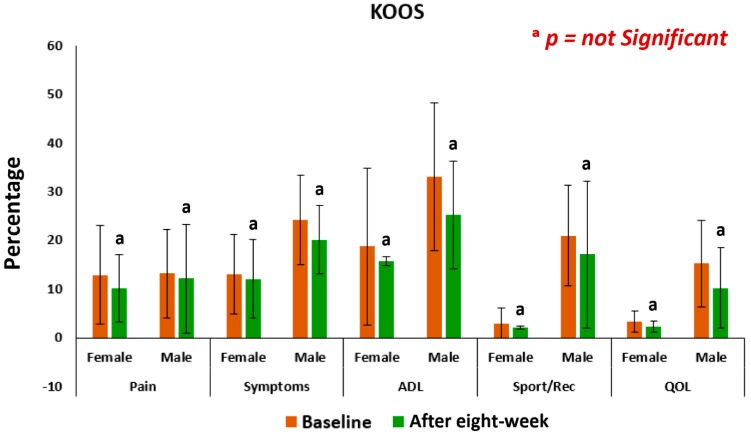
Analysis of knee-injury osteoarthritis outcome scale of 45 control subjects, female (*n* = 27) and male (*n* = 18), before and after eight-week without supplement (^a^*p* = not significant), ADL: Activities of daily living; QOL: Quality of life.

The mean levels of improvements on performance of daily activities under KPS and of the five separately scored subscales under KOOS knee survey after the eight-week treatment with supplements for female-only and male-only patients were all highly significant (*p* < 0.05) compared to the control (Figure 8 and Figure 9). The mean levels of deterioration after eight-weeks without supplements for female-only and male-only patients was not at all significant (Figure 9 and Figure 10). 

### 3.4. Improvements on Bone Health as per Radiological Images asAassessed by Kellgren-Lawrance Grading Scale

**Figure 11:**
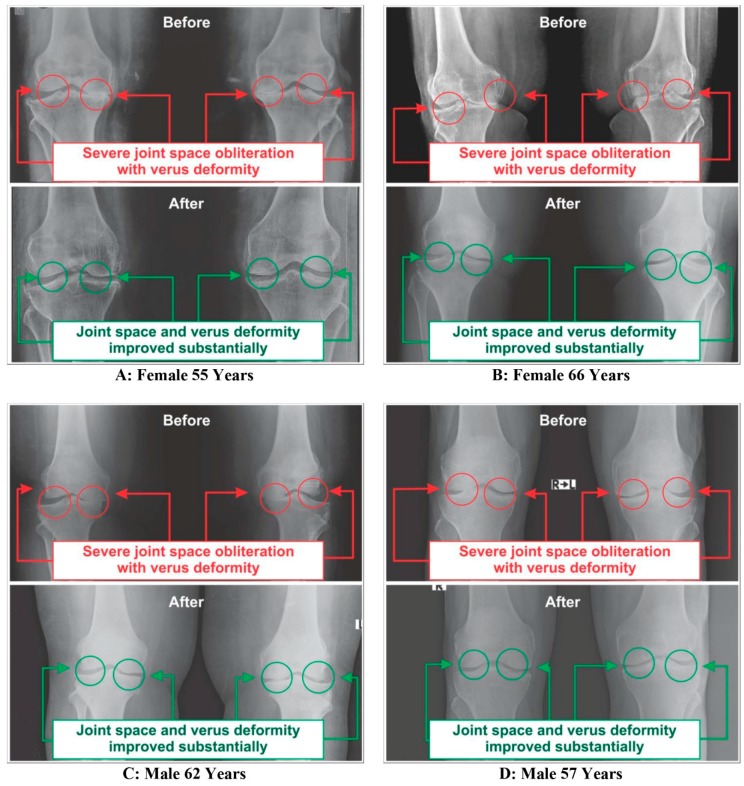
Radiological images of knee joints (two-female and two-male) showing before and after the treatment with supplement.

**Figure 12:**
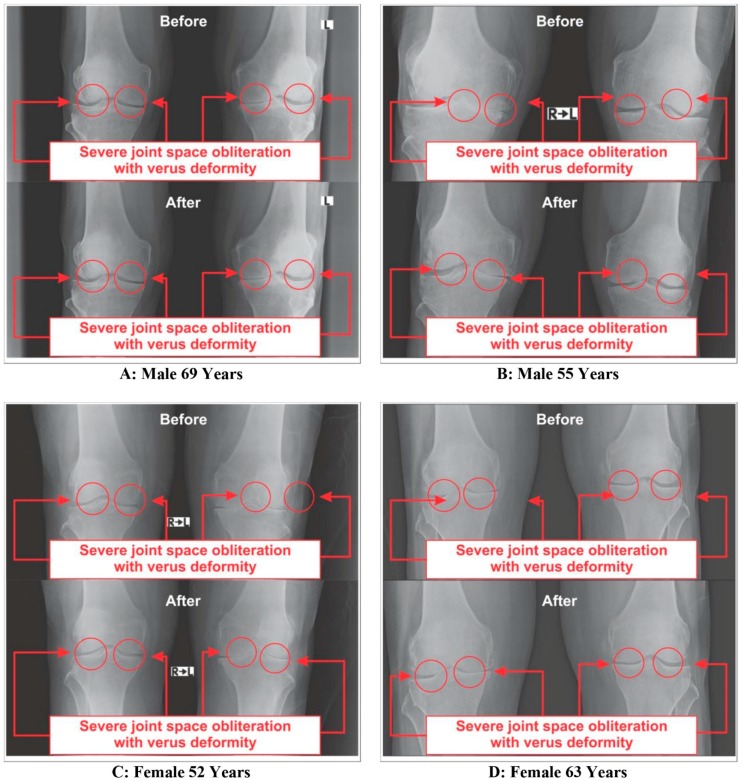
Radiological images of knee joints (two-female and two-male) showing before and after without supplement.

All the anterior-posterior (AP) views of the X-ray reports of 108 patients with OA at the baseline exhibited degenerative changes, particularly in the medial tibiofemoral compartment, with marked joint space narrowing with osteophytes and bilateral varus/valgus deformities. The AP view of X-rays for bilateral knee joints of 63 patients after eight weeks of treatment with supplements indicated substantial improvements on degenerative changes, as well as bone health and the balance of 45 patients treated without supplements had further deterioration on bone health and assessment under the KL grading scale shown in Table 4. The X-ray images of four such patients, female (*n* = 2) and male (*n* = 2), before and after the treatment with and without using supplements are depicted in Figure 11 and Figure 12, respectively. 

### 3.5. Safety and Cost Evaluation

The topics of safety and tolerability were evaluated. There were no problems with the supplement and no significant safety problems requiring the suspension of the supplement or altering compliance to the supplementation plan were observed. The use of other drugs, including physiotherapy, painkillers and rescue medication, was also decreased in the supplement group compared to control subjects (*p* < 0.05). Blood tests related to hepatic and kidney function were within the normal limits for the patients treated with the supplement after eight weeks. 

The average management cost was evaluated for the subjects using Jumpstart Nutrition^®^ bone supplement This cost was defined as an average of 100% (for the eight weeks of management) including treatment, diagnostic and loss of working days and these were reduced to 92% with range 78–92 (*p* < 0.05).

## 4. Discussion

In this pilot study, the demographic data and baseline characteristic features of 63 patients treated with Jumpstart Nutrition^®^ bone supplements and 45 patients without bone supplements, suffering with OA as confirmed with radiological and biochemical parameters, are shown in Table 1 and their gender-wise analyses are shown in Figure 1. In both the groups, women are more predominated with OA changes by 23.18% and 20% with an average age of 60.15 ± 11.25 years in the supplementary and non-supplementary groups, respectively. The results satisfy the prediction made by WHO so far as the genders as well as the age limit of people are concerned [5].

In this study, the reference value for CPR in adults was calculated based on the established lower and upper limits of calcium (8.4–10.8 mg/dL) and phosphorus (2.6–4.5 mg/dL) as 3.2–2.4 units [29,41,42,43] and for PTH and vitamin D these have been considered as 15–65 pg/mL and 30–100 ng/mL, respectively [41,42,43].

The author had previously elaborated the impact of the functional instability of parathyroid hormone and the calcium-to phosphorus ratio as risk factors for OA patients with lower levels of CPR and PTH as well as with a lower CPR level and higher level of PTH [8,9].

In general, OA treatment is based on oral and topical medicines as pain killers, gels and finally surgery [45,46,47], and to date, medical practitioners are often focused on symptomatic pain relief rather than curtailing the progression of the disease. Therefore, frequently different medications are used for temporary relief such as NSAIDs for prevention of pain and inflammation but they have tremendous side-effects such as cardiovascular and gastrointestinal problems, and corticosteroids [10,11], which may help to increase muscle strength and slow progression but can weaken bones and increase weight gain over long-term use. Other kinds of therapies such as stem cell [48] are occasionally used, which may improve the delay of progression of symptoms, thus, the quality of life may be saved temporarily.

The present results suggest that there is a close relationship of the risk factors between the bone related biomarkers (CPR, PTH and vitamin D) (Figure 2, Figure 3, Figure 4, Figure 5, Figure 6 and Figure 7 and Table 2 and Table 3) and pain with disability-related outcome measures (KOOS and KPS) (Figure 8, Figure 9 and Figure 10) in OA. The results elucidated the treatment of OA with a bone supplement especially composed of calcium, phosphorus, vitamin-K_2_, coenzyme-Q_10_, folic acid, vitamin C, boswellic acids, and curcumin, which is more efficacious in comparison with the commonly composed available supplements with vitamin D [49] or with calcium and vitamin [50] or with calcium and hydrolyzed amino acid [51] or glucosamine sulphate [52] or a combination of glucosamine sulphate and chondroitin sulphate [53] or other such combinations without vitamin-K_2_ or coenzyme-Q_10_.

The present results shows that there levels of CPR increased by 12.17% (OR: 0.07; 95% CI: 0.03, 0.29; *p* < 0.0001), PTH by 32.86% (OR: 0.36; 95% CI: 0.14, 0.94; *p* < 0.05) and vitamin D by 13.71% (OR: 1.16; 95% CI: 0.39, 3.40; *p* = 0.78) for female-only and 10.77% in CPR ( OR: 0.14; 95% CI: 0.03, 0.61; *p* < 0.05), 24.91% in PTH (OR: 0.44; 95% CI: 0.12, 1.59; *p* = 0.21) and 9.20% in vitamin D (OR: 0.59; 95% CI: 0.18, 1.90; *p* = 0.38) for male-only in the supplementary group in comparison with further decreased levels of 2.17% in CPR (OR: 3.52; 95% CI: 1.02, 12.07; *p* < 0.05), 0.72% in PTH (OR: 1; 95% CI: 0.28, 3.61; *p* = 1) and 0.52% in vitamin D (OR:1; 95% CI: 0.31, 3.22, *p* = 1) in female-only and 1.63% in CPR (OR: 1.25; 95% CI: 0.34, 4.64; *p* = 0.74), 2.33% in PTH (OR: 1; 95% CI: 0.26, 3.80; *p* = 1) and 0.27% in vitamin D (OR: 1; 95% CI: 3.22, 4.81; *p* = 1) in the non-supplementary group.

The present findings indicate a predictive risk factor through the analyses of the correlation coefficient of CPR between the pre- and post-treatment of eight weeks with the bone supplement being negatively correlated for female-only patients but positively correlated for male-only patients without significant values (*p* = 0.286 and *p* = 0.346 respectively). The correlation coefficients between the pre- and post-treatment for eight weeks with Jumpstart Nutrition^®^ for both the levels of PTH and vitamin D are highly significant (*p* < 0.001) in the case of female and male patients (Table 2).

Furthermore, it should be noted that, without increasing the strength of the muscles, the gap between the tibiofemoral and patella-femoral joints cannot be increased and thereby pain cannot be reduced. This phenomenon might be possible with the help of a coenzyme-Q_10_ supplement. It is absolutely required for muscular dysfunction associated with structural and alterations of skeletal muscle mitochondria. The dysfunction of mitochondria leads to alteration of the structure and function of organelles such as reactive oxygen species (ROS), Ca^2+^ homeostasis and apoptosis during the aging of skeletal muscle [29,54,55]. Skeletal muscle mass and muscular strength are progressively decreased due to aging, this is known as sarcopenia. Coenzyme-Q_10_ is essential for the health of virtually all human tissues (epithelial, connective, muscular and nervous) and organs, because an adequate supply of adenosine triphosphate (ATP) is dependent on most cellular functions. It is one of the most significant lipid antioxidants, which prevents the generation of free radicals and modifications of proteins, lipids and DNA [56,57,58,59,60].

At the same time, the reasons for using vitamin-K_2_ in the supplement are elaborated as follows:

- The living substance of bone is composed of a hard-outer shell and a spongy matrix of inner tissues. To meet an individual’s metabolic needs, our body releases calcium from the bone into the bloodstream allowing the bone to grow or repair from injuries during the skeleton’s remodeling [61]. It is regulated by two stages such as osteoblasts (cells that build up) and osteoclasts (cells that break down). A healthy bone structure is maintained, when the bone-forming activity is greater than the breakdown of bone.
- Osteoblasts produce inactive osteocalcin and it needs vitamin-K_2_ to become fully activated and bind calcium to make the skeleton stronger and less susceptible to fracture [28]. Again, the matrix Gla protein (MGP) of vitamin-K_2_ is a central calcification inhibitor produced by the cells of vascular smooth muscles and regulates the potentially fatal accumulation of calcium which keeps calcium from accumulating in the walls of blood vessels [62].
- However, several researchers have suggested that increased consumption of calcium supplements helps strengthen the skeleton but, at the same time, can raise the risk for heart disease as they deposited in blood vessel walls and soft tissues [63,64,65,66,67,68,69,70]. On the contrary, increased vitamin-K_2_ intake could be a means of lowering calcium-associated health risks as it is associated with the inhibition of arterial calcification and arterial stiffening [71].
- Further, it may be possible to fight osteoporosis and simultaneously prevent the calcification and stiffening of the arteries by striking the right balance in intake of calcium and vitamin-K_2_, as in the modern manufacturing processes vitamin-K_2_ content in the food supply has significantly reduced [71,72].
- Therefore, the risks for blood-vessel calcification and heart problems are significantly lowered and the elasticity of the vessel wall is increased, if at least 32 µg of vitamin-K_2_ is present in the diet [73]. Hence, 100 µg of vitamin-K_2_ per day has been recommended in the supplement.

Pain syndromes, inflammation and impaired quality of life are the major perception factors among patients with any musculoskeletal disorder, especially OA [1,3,6,7,8]. To overcome this phenomenon, boswellic acids and curcumin are added to these bone supplements [74]. As a constituent of turmeric (*Curcuma longa* L.), curcumin has been used for centuries [74,75]. This powerful antioxidant [76] is now available in a new delivery system (Jumpstart Nutrition^®^, Nanophyto Wellness Private Ltd, Kolkata, India) that improves the bioavailability of curcuminoids. In a recent, larger study, curcumin has improved OA signs/symptoms and reduced the commonly high inflammatory and oxidative values in OA patients [77], also showing an excellent safety profile. Additional analysis aims to evaluate the potential role of curcumin as a primary treatment in the complementary management of OA. Nano-curcumin and boswellic acids contained in Jumpstart Nutrition^®^ have been proven to inhibit the activation of nuclear factor kappa B (NF-kB) [74,75,78,79,80], a potent inducer of chronic inflammation [80,81].

Moreover, oral supplementation of the gum resin of *Boswellia serrata* extract (BSE) contains 3-acetyl-11-keto-β-boswellic acid (AKBBA), 11-keto-β-boswellic acid (KBBA), β-boswellic acid (BBA), and 3-acetyl-β-boswellic acid (ABBA) [82]. Biologically active constituents of BSE, namely, β-boswellic acid (BBA) and 3-acetyl-11-keto-β-boswellic acid (AKBBA), act synergistically to exert anti-inflammatory/anti-arthritic activity to reduce joint pain and improve physical functional ability in patients with OA of the knee [83]. Researchers have proved that the oral supplement of BSE can significantly reduce the potential inflammatory marker, serum levels of high-sensitive C-reactive protein and improve the knee joint gap and reduce the osteophytes (spur), associated with OA [84]. Boswellic acids also inhibit lipopolysaccharide-mediated TNF-α induction in monocytes by direct interaction with IκB kinases [80].

Again, whey protein or soy protein is considered to be the highest quality natural protein [85] and the bioactive substances of whey protein or soy protein, such as hormones, growth factors and cytokines, which can have an important physiological role of regulating cell growth in both normal cells and tumor cells by suppressing proliferation or enhancing apoptosis [34,35,86].

In the present study, it indicates that all the internationally-acclaimed pain-related parameters under KOOS and KPS are in much more favorable positions (decreasing levels of pain activities and increasing levels of life style) in the patients in the supplementary group at the end of the eight-week treatment protocol, compared to control group without the supplement (Figure 8 and Figure 10). Therefore, Jumpstart Nutrition^®^ exerts a significant anti-inflammatory action in these patients, leading to a reduction in symptoms.

Moreover, the results as shown in Table 4 and Figure 11 indicate the definite improvements on reducing osteophytes, joint space narrowing, sclerosis, bony deformities of knee-joints of the OA patients treated with Jumpstart Nutrition^®^ in the supplementary group, as assessed by the KL grading scale.

Finally, this was a small-scale, independent, pilot, registry study; the evaluation product was not prescribed but recommended. In this way, the registry is more similar to real, practical conditions than most clinical studies, which artificially select groups of patients in very controlled conditions, often not comparable to an epidemiological reality.

This type of supplement may be particularly suited for emerging countries and when expensive sponsorships for brand products are not available.

This observational study in otherwise healthy, chronic OA patients indicate that the administration of Jumpstart Nutrition^®^ bone supplement improves outcomes in comparison with the standard management.

Jumpstart Nutrition^®^ bone supplement decreased the need for further medical attention, with a consequent reduction in medical costs.

At the moment, observational and supplement studies or clinical trials on boswellic acids and curcumin in standardized pharmaceutical forms are limited [30,31,32,75,87,88]. 

The focus in this pilot registry study and in previous evaluations of OA patients was mainly on the safety profile and on the efficacy of Jumpstart Nutrition^®^ in reducing and controlling pain, in the management of costs and the use of other products, and in improving quality of life. Considering the tremendous opportunity for using safe products of a natural origin in pharmaceutical standards (PS supplements), particularly in subjects in remission or non-acute phases, Jumpstart Nutrition^®^ bone supplement could be an important management option, also considering the large social cost of managing these patients whose number is growing with increasing age [74]. 

This supplement registry indicates that the use of Jumpstart Nutrition^®^ supplementation in OA (which may be used even without prescription for safe self-medication) is effective and has limited costs.

Jumpstart Nutrition^®^ especially combined with vitamin-K_2_, coenzyme Q _10_, vitamin D_2_, vitamin C, boswellic acids and curcumin mixed with protein powers of soy and whey seems to be rapidly effective even in comparison with other bone supplementary products, as mentioned above, on the signs/symptoms of OA. It acts rapidly in a large number of patients [89], including subjects with a main inflammatory component and those with a degenerative component (associated to different types and levels of pain) as well as formation of calcium and phosphorus metabolism along with the maintenance of appropriate levels of PTH and vitamin D in the serum.

However, this study has several important limitations. Firstly, we have taken a small sample size and have not yet confirmed whether these results are biased. Secondly, the present study relied predominantly on X-ray based determination of OA, but higher resolution magnetic resonance imaging (MRI) modalities may prove to be a more sensitive measure of cartilage pathology and a stronger clinical correlation than symptoms and radiographic measures. Thirdly, patients are restricted to treat with the supplement those who are suffering with following disorders: intolerant of milk products; wounds or any types of chronic skin and infectious diseases; parallel multiple drug dependence for concomitant diseases or risk conditions requiring drug treatment including psychiatric diseases; a history of cancer including caranomatosis and granulocytic leukemia; a history of chronic liver, and kidney diseases, and also patients who did not agree to give blood sample, maybe due to drugs/alcohol addiction, pregnancy and such other reasons and a physical evaluation and /or attend weekly follow-up visits.

## 5. Conclusions

This supplement registry study suggests that Jumpstart Nutrition^®^ can be considered an effective bone nutrition supplement for the management of OA patients for the improvement of risk factors such as CPR, PTH, vitamin D (Figure 2, Figure 3 and Figure 4, Table 2 and Table 3) and internationally approved outcome measures along with functional activities (Figure 8 and Figure 9) confirming findings with biochemical parameters and knee joint radiographic images correlated with the Kellgren-Lawrance grading scale (Figure 11 and Table 4).

Further research is suggested to be undertaken for the evaluation of inflammation, muscular dystrophy, connective tissue damage and skeletal muscle damage with the help of analyses of suitable biomarkers such as C-reactive protein, creatine kinase-muscle and aldolase-A and anatomical parameters such as range of motions, waist to-hip ratio and body mass index by using Jumpstart Nutrition^®^ bone supplement in other musculoskeletal diseases such as rheumatoid arthritis.

## Figures and Tables

**Table 1:** Demographic data and baseline characteristics of the study subjects.

Characteristic	Control Group		Experimental Group	
	Female	Male	Female	Male
No of subjects (%)	27 (60.00)	18 (40.00)	39 (61.90)	24 (38.10)
Mean age (SD) in years	60.89 (11.37)	61.38 (10.21)	60.78 (11.13)	61.12 (13.68)
Mean weight (SD) in kg	71.71 (5.05)	71.32 (5.11)	71.67 (6.12)	70.77 (6.78)
Mean height (SD) in meter	1.55 (0.92)	1.58 (0.88)	1.56 (0.93)	1.57(0.98)
Mean BMI (SD) in kg/m²	29.85 (6.94)	28.57 (6.64)	29.43 (7.11)	28.71 (6.32)
Mean symptom duration in years (SD)	6.89 (1.90)	7.78(1.78)	6.12 (2.11)	7.26 (2.01)
**Indian ethnic group (%)**				
Bengali	8 (29.63)	5 (27.78)	14 (35.90)	6 (25.00)
Gujrati	3 (11.11)	2 (11.11)	6 (15.38)	3 (12.50)
Marwaree	5 (18.52)	2 (11.11)	4 (10.26)	4 (16.67)
Marathi	3 (11.11)	2 (11.11)	3 (7.69)	3 (12.50)
Tamil	2 (7.41)	2 (11.11)	2 (5.13)	2 (8.33)
Punjabi	2 (7.41)	1 (5.56)	4 (10.26)	2 (8.33)
Shindhi	1 (3.70)	2 (11.11)	3 (7.69)	2 (8.33)
North East India	3 (11.11)	2 (11.11)	3 (7.69)	2 (8.33)
**Dietary habits (%)**				
Vegetarian	12 (44.44)	10 (55.56)	28 (71.79)	10 (41.67)
Non-vegetarian	15 (55.56)	8 (44.44)	11 (28.21)	14 (58.33)
**Other habits (%)**				
Smoking	3 (11.11)	9 (50.00)	2 (5.13)	5 (20.83)
Drinking alcohol	7 (25.93)	11 (61.11)	6 (15.38)	11 (45.83)
Drinking tea and coffee	12 (44.44)	16 (88.89)	18 (46.15)	12 (50.00)
Chewing tobacco	2 (7.41)	4 (22.22)	3 (7.69)	4 (16.67)
**Analysis of radiological reports (%)**				
KOA in right knee with osteophytes	10 (37.04)	8 (44.44)	12 (30.77)	7 (29.17)
KOA in left knee with osteophytes	17 (62.96)	10 (55.56)	27 (69.23)	17 (70.83)
**Work status (%)**				
Employed fulltime	2 (7.41)	7 (38.89)	3 (7.69)	8 (33.33)
Employed part time	1 (3.70)	2 (11.11)	2 (5.13)	2 (8.33)
Housewife / Home- maker	20 (74.07)	0 (0.00)	27 (69.23)	0 (0.00)
Retired	2 (7.41)	6 (33.33)	4 (10.26)	10 (41.67)
Self employed	2 (7.41)	3 (16.67)	3 (7.69)	4 (16.67)
**Marital status (%)**				
Single	2 (7.41)	1 (5.56)	2 (5.13)	1 (4.17)
Married	17 (62.96)	14 (77.78)	28 (71.79)	20 (83.33)
Separated	2 (7.41)	1 (5.56)	2 (5.13)	1 (4.17)
Divorced	1 (3.70)	2 (11.11)	2 (5.13)	2 (8.33)
Widowed	5 (18.52)	0 (0.00)	5 (12.82)	0 (0.00)
**Multiple complaints or comorbidities (%)**				
Constipation	17 (62.96)	10 (55.56)	27 (69.23)	16 (66.67)
Acidity and reflux	12 (44.44)	7 (38.89)	16 (41.03)	18 (75.00)
Insomnia	10 (37.04)	8 (44.44)	18 (46.15)	8 (33.33)
Varicose veins	8 (29.63)	6 (33.33)	14 (35.90)	11 (45.83)
Urinary incontinence	13 (48.15)	7 (38.89)	19 (48.72)	18 (75.00)
Crepitus during knee flexion	14 (51.85)	8 (44.44)	21 (53.85)	19 (79.17)
Morning stiffness (<30 min.)	17 (62.96)	10 (55.56)	18 (46.15)	14 (58.33)
**Measures taken to diminish pain and inflammation (%)**				
Kneecap uses	24 (88.89)	16 (88.89)	34 (87.18)	18 (75.00)
Lumbar belt uses	5 (18.52)	2 (11.11)	4 (10.26)	2 (8.33)
Paracetamol and NSAID use	26 (96.30)	16 (88.89)	37 (94.87)	21 (87.50)
Arthrocentesis (four months ago)	11 (40.74)	7 (38.89)	8 (20.51)	5 (20.83)
Use of hyaluronic acid injection	8 (29.63)	7 (38.89)	6 (15.38)	5 (20.83)
Use of corticosteroid injection	6 (22.22)	8 (44.44)	7 (17.95)	6 (25.00)
Massage with herbal or other gels	18 (66.67)	20 (111.11)	25 (64.10)	17 (70.83)
Homeopathic treatment	19 (70.37)	16 (88.89)	19 (48.72)	18 (75.00)
Ayurvedic treatment	21 (77.78)	17 (94.44)	20 (51.28)	20 (83.33)
Stick/walker use	18 (66.67)	11 (61.11)	18 (46.15)	10 (41.67)
**Supplements taken to reduce pain or improve fitness (%)**				
Calcium with vitamin D	11 (40.74)	10 (55.56)	32 (82.05)	19 (79.17)
Vitamin D injection	8 (29.63)	7 (38.89)	14 (35.90)	6 (25.00)
Glucosamine	6 (22.22)	8 (44.44)	9 (23.08)	4 (16.67)
Glucosamine and chondroitin	5 (18.52)	4 (22.22)	7 (17.95)	3 (12.50)

**Table 2:** Analysis of correlation coefficients of biomarkers between baseline and end of eight-week treatment with supplement.

Comparison of Two Biomarkers	Female (*n* = 39)		Male (*n* = 24)	
	r-Value	*p*-Value	r-Value	*p*-Value
CPR^b^-Vs-CPR^t^	−0.175	0.286	−0.201	0.346
PTH^b^-Vs-PTH^t^	0.496	0.001	0.664	0.0004
Vit.D^b^-Vs-Vit.D^t^	0.786	0.000	0.390	0.000

CPR^b^: level of calcium -to-phosphorus ratio at the baseline; CPR^t^: level of calcium -to-phosphorus ratio at the end of eight-week with supplement; PTH^b^: level of parathyroid hormone at baseline: PTH^t^: Level of parathyroid hormone at the end of eight-week with supplement; VitD^b^: level of vitamin D at baseline; VitD^t^: Level of vitamin D at the end of eight-week with supplement; *n* = number of subjects.

**Table 3:** Analysis of coefficient of variance of control and experimental groups.

Biomarker	Control Group without Supplement (*n* = 45)				Experimental Group with Supplement (*n* = 63)			
	Baseline		After Eight-Week		Baseline		After Eight-Week	
	Female	Male	Female	Male	Female	Male	Female	Male
CPR (%)	26.73	17.6	20.58	19.31	6.91	6.5	9.75	7.41
PTH (%)	41.67	60.72	41.49	58.31	41.6	40.83	20.53	18.36
Vitamin D (%)	43.13	49.37	43.45	49.67	43.6	30.89	33.12	29.35

CPR = Calcium -to-phosphorus ratio; PTH = Parathyroid hormone; *n* = number of subjects.

**Table 4:** Kellgren-Lawrence (KL) Grading Scale for Knee-osteoarthritis of experimental and control groups.

Knee Joints	Gradation	Control Group (*n* = 45)				Experimental Group (*n* = 63)			
		Baseline		After Eight-Week		Baseline		After Eight-Week	
		Number	%	Number	%	Number	%	Number	%
**KOA (Rt. knee)**	Grade-0	None	None	None	None	None	None	None	None
	Grade-1	None	None	None	None	None	None	4	6.35
	Grade-2	None	None	None	None	None	None	8	12.70
	Grade-3	23	51.11	21	46.67	28	44.44	30	47.62
	Grade-4	22	48.89	24	53.33	35	55.56	21	33.33
**KOA (Lt. knee)**	Grade-0	None	None	None	None	None	None	None	None
	Grade-1	None	None	None	None	None	None	3	4.76
	Grade-2	None	None	None	None	None	None	7	11.11
	Grade-3	19	42.22	17	37.78	27	42.86	31	49.21
	Grade-4	26	57.78	28	62.22	36	57.14	22	34.92

Grade 0: no radiographic features of OA are present; Grade 1: doubtful joint space narrowing (JSN) and possible osteophytic lipping; Grade 2: definite osteophytes and possible JSN on anteroposterior weight-bearing radiograph; Grade 3: multiple osteophytes, definite JSN, sclerosis, possible bony deformity; Grade 4: large osteophytes, marked JSN, severe sclerosis and definite bony deformity; *n* = number of subjects, KOA: Knee osteoarthritis.
